# The anatomy of crisis

**DOI:** 10.1080/17482631.2024.2416580

**Published:** 2024-10-17

**Authors:** Helena Roennfeldt, Nicole Hill, Louise Byrne, Bridget Hamilton

**Affiliations:** a Centre for Mental Health Nursing, Department of Nursing, University of Melbourne, Melbourne, Australia; b Department of Social Work, University of Melbourne, Melbourne, Australia; c School of Management, RMIT University, Melbourne, Australia; d Program for Recovery and Community Health, Department of Psychiatry, Yale School of Medicine, New Haven, CT, USA

**Keywords:** Crisis, phenomenology, lifeworld, mental health, suicide

## Abstract

This phenomenological study deeply explores the individual and collective lived experience of a mental health crisis. A Lifeworld approach provided the entry point to deeper insights into the *anatomy of crisis* as the embodied emotional, physical, cognitive, and spiritual nature of crisis. Findings uncovered rich descriptions of mental health crises and how the crisis was encountered in a shattered sense of self and relational challenges in the context of receiving crisis care. Overall, the study revealed an embodied understanding of crisis that offers practical direction in providing crisis care that is more attuned to lived experience.

Behind your thoughts and feelings, my brother, stands a mighty commander, an unknown sage—he is called Self. He lives in your body, he is your body. (Nietzsche, 1961 [1883], p. 62)

## Background

The expression “I’m in crisis” may seem straightforward, but how is a crisis subjectively experienced? And how does it specifically relate to the context of accessing crisis care? A state of crisis is not self-evident but related to the subjective experience of the person and the context in which it is being expressed. Traditionally, a crisis was defined as psychological disequilibrium in response to external events (Caplan, 1964). More recently, the term mental health crisis has been used to refer to a complex type of crisis that is associated with a psychiatric diagnosis (Rosen, 1997). However, the term crisis lacks consistency in mental health literature (Bonynge et al., 2005; Callahan, 1994; Hobbs, 1984; Hogan & Goldman, 2021; Paton et al., 2016), and there has been a shift towards defining mental health crisis synonymously with a psychiatric emergency, using criteria for involuntary hospitalization, namely as a “situation in which a person’s behaviour puts them at risk of hurting themselves or others and/or prevents them from being able to care for themselves or function effectively” (Brister, 2018, p. page 5). Using this definition, the assessment of crisis is frequently based on standardized psychiatric criteria and triage processes that prioritize the risk of harm to self or others (Yeager & Roberts, 2015).

In contrast, first-person accounts of crisis have challenged these views, instead highlighting the emotional distress and interpersonal, existential nature of crisis (Bögle & Boden, 2019; Borg et al., 2011; Gullslett et al., 2014, 2016; Winness et al., 2010). These studies reflect mental health crisis as a subjective term describing the excruciating emotional effect that life situations can have on an individual (Gullslett et al., 2016; James & Gilliland, 2016), which are challenging to frame within diagnosis or standardized criteria of risk (Lumley et al., 2011). However, this literature has tended to have a limited focus on crises associated with specific diagnoses, such as crises associated with psychosis (Bögle & Boden, 2019) or related to exiting service users receiving crisis care within a specific service (Borg et al., 2011; Gullslett et al., 2014).

Developing an understanding of crisis is significant given that in many developed countries, there is an increase in people presenting with mental and emotional distress to general hospital emergency departments (ED) (Alakeson et al., 2010; Roggenkamp et al., 2018). Within this clinical environment, a pathology-oriented perspective is often assumed (Adams, 2012; Lloyd‐Evans et al., 2018; Murphy et al., 2012), and there is a scarcity of crisis support available for people without a diagnosis of mental illness or with undefined distress (Rhodes & Giles, 2014). In response to the increasing demand placed on ED, there are policy imperatives in Australia and many countries internationally to improve access to mental health crisis care (Vacher et al., 2023).

Against this backdrop, this study takes a step back to understand the lived experience of crisis from people who have accessed emergency departments (ED), crisis phone lines, or crisis alternatives. The study is grounded in a hermeneutic phenomenological and Lifeworld approach involving rich description, interpretation, and reflection on subjective experience (Heidegger et al., 1962). Phenomenology honours the body as the opening to the world and vehicle for understanding lived experience (Merleau-Ponty, 2004), while Lifeworld provides a means to organize lived experience using dimensions of temporality (experience of time), spatiality (experience of space), embodiment (experience within a physical body), sociality (relationship to others), project (experience of purpose and meaning), self (experience of the concept of self and identity) and discourse (thoughts and language) (Dahlberg et al., 2009; Finlay, 2011; Galvin & Todres, 2013). A Lifeworld approach was employed to give an embodied and contextual understanding of a crisis, acknowledging our experiences as inseparable and interrelated to the world (P. Ashworth, 2003; Heidegger, 2010; Merleau-Ponty & Smith, 1962; Sharma et al., 2009).

Lifeworld approaches offer a counterpoint to a reductionist view of mental illness that is based on a focus on the brain, obscuring other human and physical dimensions of experience (Stein et al., 2022). Accordingly, this study is situated alongside more critical phenomenological examinations of the psychiatrization of human experiences (Zahavi & Loidolt, 2022). Previous literature identifies a need for more humanizing approaches within crisis care, with reports that personhood can be obscured and people do not often feel met as humans (Jenkins et al., 2023). Likewise, there is considerable evidence that individuals with mental health concerns face discrimination compared to people presenting with physical emergencies within ED settings (Da Silva et al., 2020; Duggan et al., 2020). A recent review of subjective experiences of ED highlighted the need to focus on social, physical, emotional, and psychological dimensions within an individualized approach to crisis care (Roennfeldt et al., 2021). It is proposed that the Lifeworld is an entry point to enhance humanly sensitive crisis care by attending to experiences of “what it is like” to be in crisis (Galvin et al., 2020; Todres et al., 2007).

This study expands on previous understandings of crises by deeply exploring the lived experiences of people who have accessed crisis services and using rich descriptions to reveal the embodied crisis experience—the *anatomy of a mental health crisis*. By centring lived experience voices, this study seeks to produce research informed by people most impacted (Beresford & Carr, 2016) to mobilize more robust person-centred approaches within crisis care. Further, the first author openly identified as having personal experiences of crisis and drew on insider research, which serves as a means for epistemic justice and liberation, especially in phenomenological research, as it prioritizes the voices of those with first-hand experience over pre-existing theory or ontology (Fricker, 2007).

## Method

### Study design

The study uses hermeneutic phenomenological research to explore the nature of human experience as embodied and embedded within the participant’s Lifeworld (P. D. Ashworth, 2016; Finlay, 2014; Heidegger, 1999). Lifeworld situates participants’ lived experiences within their life situations, the broader cultural and historical context, and the specifics of the research situation (Finlay, 2014; Thompson, 2018). Through “bridling” what is already known and challenging assumptions, the researcher allows phenomena to reveal themselves (Finlay, 2014; Heidegger, 1999).

The study was approved by the University of Melbourne Human Research Ethics Committee, established as per the National Statement, and in accordance with the principles of the Declaration of Helsinki (World Medical Association, 2013). Participants were given both verbal and written information before consenting to participate. In accordance with ethical approval, the names of the participants given in the findings are pseudonyms or participants’ names, depending on preference.

### Participants

Recruitment was initiated via email and social media to Australian mental health Consumer and Peer networks. The inclusion criteria for participation were: (a) self-identified as having experienced a mental health crisis AND b) in response to this crisis, sought support at an emergency department, crisis phone line, or alternative crisis service. Potential participants were contacted to confirm they met the inclusion criteria. Purposeful selection of study participants (over 18 years) was undertaken to attempt to gain a representation of different crisis care experiences, and recruitment continued until there was a representation of people who had received crisis care across ED, crisis phone lines, and crisis alternatives.

### Data collection

Data collection involved in-depth interviews to give insight into the participants’ felt experiences through verbal accounts and descriptions of their embodied experiences. Participants were asked to describe their sensory experience of crisis and their understanding of crisis in their lives, which was apparent in questions such as: How is it experienced emotionally? How is it experienced physically? What does it mean for identity and a sense of self? And how does it relate to what you care about and find meaning in the world?

The first author conducted all interviews and was explicit about their lived experience, openly disclosing at the beginning of the interview. This was seen as enhancing empathy and trust, and having someone who shares a common experience, language, and identity with research participants has been shown to help build rapport, encourage more open participation, and yield more comprehensive data (Dwyer & Buckle, 2009). Interviews were audio-recorded and transcribed verbatim. All participants provided informed consent for the recording and transcription of interviews prior to their inclusion in the study. The majority of interviews were conducted via the Zoom video platform, with two interviews each occurring by phone and in person. The interviews had a mean duration of 80 minutes (SD = 42).

### Data analysis

A hermeneutic-phenomenological approach recognizes that analysis involves interpretation as the researcher tries to make sense of the participant’s meaning-making (Churchill, 2018; Finlay, 2002). Interviews were analysed individually and then across the data set to identify common themes. The Lifeworld dimensions provided a final theoretical overlay and understanding of the embodied crisis experience. Table I provides a detailed description of the Lifeworld dimensions.

**Table I.** Lifeworld dimensions.

Lifeworld dimension	Description
Mood	How does the experience affect our emotions? Lived experience is colored by mood which in turn colors all dimensions of the Lifeworld. Mood has organizing power and is a great motivator or demotivator (Todres & Todres, 2007)
Embodiment	How does it feel physically within our bodies? Embodiment is also about how we experience our own bodies, including gender, disability, and emotions. A body is a ‘being in the world,’ and in phenomenological research, the distinction is made between the objective and subjective body (Merleau-Ponty, 2004).
Discourse	What terms are used to describe crisis? The terms we use to describe and give meaning to our experience and how we make sense of our world (P. Ashworth & Chung, 2007).
Self	What does it mean for sense of self? Includes how we experience our understanding of self, identity and agency (P. Ashworth, 2003).
Temporality	How is time experienced? Temporality refers to a sense of time, and time has a quantitative and qualitative sense (Van den Berg, 1972).
Spatiality	How is our experience affected by our physical and relational space? What is the meaning given to the surrounding environment and places we experience crisis? The meaning of closeness and distance within interactions with others affect our lived experience. It is not merely the physical space but the interpretive meaning of space (Soltani & Kirci, 2019).
Sociality	How does the experience affect relationships with others? How we relate to others and how others relate to us is critical to understanding our experience and sense of being in the world. The individual experience cannot be fully understood without considering the social dimension (Heidegger, 1977).
Project	How does the experience relate to our capacity to find purpose and meaning and do the activities we are committed to and consider important in life (Finlay, 2013)?

The first author initially coded the data and engaged in an ongoing discussion of potential emerging themes with the second and last authors, as well as repeated refinement of codes. The analysis drew on the iterative phenomenological research approach by Finlay, encompassing: (a) embracing the phenomenological attitude, (b) entering the lifeworld of participants through descriptions of experiences, (c) dwelling with implicit meanings, (d) explicating the phenomenon, and (e) integrating frames of reference (Finlay, 2013).

Within the analysis, metaphors were selected to deepen the understanding of the Lifeworld and how participants gave meaning to their embodied subjectivity based on metaphorical language (Todres et al., 2007). Metaphors drew on symbols and images, aiding the uncovering of pre-reflective and sensory experiences that were not easily verbalized (Boden & Eatough, 2014).

### Reflexivity

For hermeneutic phenomenological researchers, reflexivity involves critically reflecting upon and examining the influence of any personal bias or assumptions that can potentially affect the representation of the research findings (Finlay, 2002; Merleau-Ponty, 2002). Within the study, reflexivity involved journaling and regular meetings with the authorship team to examine emerging themes (Finlay, 2011). Researcher subjectivity and interpretation are valued in hermeneutic phenomenological research but need to be grounded in participants’ accounts; therefore, continual reflexivity was needed to separate pre-existing assumptions and interpretations from the participants’ descriptions (Finlay, 2009; Finlay, 2011).

## Results

### Participant characteristics

The final sample consisted of 31 people with a mean age of 42 (SD = 13). Most participants identified as female (20), with a smaller number identifying as male (7), non-binary (2) and transgender (2). The final sample did not include five interested individuals who approached regarding participation. Three decided not to participate for personal reasons, and two did not attend the interview. The demographic questions revealed that most participants identified as having accessed ED (*n* = 30), 25 participants had used crisis phone lines, and 15 participants had accessed crisis alternatives. Participants explained their crisis as associated with self-injury, suicidality, psychosis, eating disorders, and overwhelming emotions resulting from grief, past trauma, or other life situations.

### Embodied experience of crisis

During the analysis, it became clear that the themes corresponded to Lifeworld dimensions of mood, embodiment, discourse, project, sociality, spatiality, temporality, and self. Participants’ accounts of crisis could be seen as comprised of core interrelated embodied elements that extended to the relational context of receiving crisis care. Themes are presented alongside the corresponding Lifeworld dimension provided in brackets.

The core elements of the embodied crisis experience are initially illustrated with supporting extracts before incorporating the relational aspects during crisis care. Suicide features strongly across participant experiences as an escape from the excruciating embodied experience. Spirituality is included as part of the project dimension within participants’ Lifeworld, and the potential for transformation also counterpoints the negative beliefs about crisis. The embodied experience of crisis begins by detailing the emotional aspects of crisis.

#### Intense and overwhelming emotional pain (mood)

Participants described the crisis as intense and overwhelming emotions, such as “shame,” “terror,” “hollowness,” and “loneliness.” The following quote captures this emotional intensity.

The horror. I use the word horror because that’s a huge emotional word. And it’s hard to express how monstrous the pain, the fear, the guilt, the horror. Clare

Emotions were experienced as “gut-wrenching” and bundled together as “emotional pain.”

Most of the time, I was just in this extreme crisis where I was feeling like so emotionally overwhelmed. And, just the emotional pain was so intense. Cathy

Emotions were also characterized using evocative metaphors, with “darkness” and “a sensation of being on fire.” Participants also used comparisons to convey their emotional pain, such as “feeling like someone will kill your loved ones.” The result was a sense of desperation and hopelessness.

It feels like sheer terror. It is like whichever way you turn, you can’t see an ounce or even a glimpse of hope of changing your position in how it is or just any of it. You’re in a black hole. But it’s more than that. It’s kind of like someone telling you that they are gonna kill your loved one. Not so much yourself, but your loved one and you can’t do anything about it. Di

The intense emotional experience was associated with a desire to escape the emotional pain, expressed as thoughts of suicide.

And with these emotions coming out, the emotions just kept getting bigger and bigger and bigger. It just got to the point that it was just too much, and I just thought, I don’t want to be here. I really just don’t want to keep going through this. It’s just out of control. Leanne

Emotions were the most compelling in participants’ crisis accounts, and a crisis was inextricably linked to emotional pain. Therefore, the lived experience of the crisis was coloured by mood; consequently, emotions coloured all other dimensions of the Lifeworld. Emotions were visceral and interwoven within physical experiences of crisis.

#### The intolerability of being in my skin (embodiment)

The experience of crisis was experienced within the body, and many participants talked about physical experiences of being in crisis, including sensations of “skin itching,” “agitation,” “exhaustion,” and feeling “physically sick.” The physicality of crisis is exemplified in the following quote.

Crisis became embodied for me and was definitely something that was restrictive. And yeah, almost like coming to a brick wall and it’s just like, that’s how I felt as well inside myself, like very tense and quite distressed. So, the physical pain and … it led to lots of tension. And I would shout, and I would make noises like wail and moan, for instance. Darcy

The physical sensations were often described as excruciating, and participants struggled to be in their bodies, describing an urgency to escape and alleviate their distress.

You know, physically, I feel like this energy in my body that needs to escape, and it’s like, you know, popcorn in a bag, just like bursting around with nowhere to go. And that’s really uncomfortable. And there’s a strong sense of urgency of just needing to get it out, and often that involves some kind of self-harm or a suicide attempt. Tyler

In contrast, some participants described the relief that they felt in their bodies at the point of suicide, knowing that there was a way to escape their emotional pain.

The greatest feeling that I had in my body was a beautiful, calm confidence. I’d made up my mind that this was what I needed to do. I’d sought out help, which actually hadn’t helped. And it was like, ah, okay, I’ve had a gut full of this. My body was like, ah, thank God, it might actually end, the pain, the fear, the self-disgust, all those things. It was like I was waiting for the relief of opening up the escape hatch and escaping from the damaged submarine. It was like a warm feeling of saying, God, I finally got to the point where I can leave this awful life. See what I mean? it’s not, it’s not what most people think that you get terribly hysterical and go and get the shotgun and bang. Sometimes it’s quiet. It’s a long-term thing. Clare

For many participants, the emotional and physical sensations were entangled and hard to separate.

If you’ve ever experienced where you feel like just every nerve is so shallow in your skin that everything is an irritation like physically and emotionally. It’s almost like you’re on that knife edge. And it’s hard to describe in words but that whole sense of it’s just pain that’s physical and emotional. Justin

The embodied experience was often less accessible to participants’ recall and required additional time to remember what the experience of crisis felt like in their bodies. Yet, with reflection, participants readily identified crisis as manifesting physically. The sensations were uncomfortable and mirrored the intolerability and emotional overwhelm, which in turn affected their thinking.

#### Constant negative voices in my head (discourse)

The experience of crisis included the loss of logical thinking and persistent negative thoughts, which one participant described as “like a scratched record that just goes around and around and around” (Tyler). Similarly, participants described the impact of the constant negative voices in their heads.

Constantly having this fog over my head, you know, like feeling lost and worthless, there’s so many other things, shame. Just you know they are all sort of painful stuff that sits in your gut, you know, and the constant voices in your head, the negative voices and negative talk. Corey

These thoughts were, at times, so distressing that they felt intolerable.

I think sometimes it’s like, it’s almost like my head is so full of stuff, thoughts, and its mainly thoughts, I guess, but it gets to the point where I don’t even know what it feels like, there’s so much in there that I don’t even know what I’m thinking anymore. And I just want it to stop. Yeah, it’s like it gets to the point where I just, I’m just like, I’m out. I guess I imagine it as like, there’s all these voices. Like when I say that I don’t mean that I hear voices, but that it’s like, there’s so many different voices. It’s like if you’re standing in a shopping mall, and it’s really loud because there are people everywhere talking. That’s what it’s like. Amie

The crisis also affected participants’ capacity to communicate their experience in a way that could be understood by others, and this included the inability to find words to express their experience.

I couldn’t communicate to people about what was happening, whether it was physically or emotionally or wherever. Alina

For many participants, the word crisis itself was often a helpful way to understand their experience and the significance of this experience in their lives.

Crisis is good. Crisis is significant. I like it. It resonates. And it’s not, yeah, I don’t think it’s a bad way of framing it. Andy

Language and meaning making during a crisis were restricted by the accessibility of words to convey experiences. Participants described overwhelmingly negative and repetitive thoughts that were closely linked to emotions and self-identity.

#### Shattered sense of self (self)

The culmination of the embodiment of crisis was felt as impacting participants’ identity and sense of self, and participants spoke of “self-hatred,” “self-loathing,” and an “intolerability of being in yourself.”

Sometimes, it’s the belief that I shouldn’t be here and that I’m toxic, and that I’m going to be toxic to everyone around me, that I’m some kind of cancer and I’m going to leach out and be toxic to the rest of the world. And I should just die. Amy

Other participants reported a loss of self or a misremembering of who they were.

It feels like a misunderstanding or misconstruction of reality, and also a kind of miss remembering of who I am. Misidentifying who I am. Andy

Sense of self was also reflected in how participants understood the impact of the crisis experience on their personhood.

You then become invisible. You’re an invisible person. So, you can’t, like you’re different. You’re not the same. You know, you’re, you can’t be seen the same as somebody else. Blair

Participants provided insights into how crises shattered their sense of self and negatively impacted their experience of their place in the world. The heightened sensory experience and encounters with others during crises contributed to perceptual changes within time and space.

#### Urgency and distortions of time(temporality)

Time was often perceived differently during a period of crisis. In a similar way to Tyhurst (1957), one participant described the blurring of time, as the past was felt to come back to meet the present.

So yeah, I can feel my body tensing up now, even. And of course, tears are welling up. And yeah, I guess my mind kind of starts racing but then in a way it kind of goes distant as well. Like, yeah, it almost goes, like it tries to go back in time, I guess. Chrystal

The crisis resulted in a greater sense of urgency to alleviate the emotional pain and reduced the perception of safety.

Normally, when I am in that sort of acute distress, it’s that really heightened impulsive crisis. And within four hours, I’ll be suicidal. I’ll be at the depth of despair. Fiona

The intensity of the crisis was noted more at night, representing increased isolation.

Sometimes, it is too hard to actually talk to someone face to face about that kind of thing because, quite often, it happens at nighttime anyway, when there’s no support worker or psychologist or anything like that available. Chrystal

Considerations of time related to the point of encounter with others in receiving crisis care.

I feel like I am wasting the nurse’s time when I’m in the ED because I know they’ve got other patients who are physically unwell, and they need monitoring. But because I’m there, they need to come and check on me and do tests and stuff as well. So, I almost feel like I’m wasting their time. Chrystal

A crisis created a sense of desperation and immediacy in needing support. The subjective experience of time was complex and relational in that participants noted the perception of wasting others’ time.

#### No safe space (spatiality)

The embodied experience of the physical space of receiving crisis care within the ED had a profound impact, leaving participants feeling othered, without privacy, and trapped.

It was a room with no window and a corridor that would have been two or three metres long. And to be honest, in terms of like, a vivid memory sort of thing, like it feels like that was like a tunnel. It really feels like a walk down a tunnel. It felt very much like, like almost walking into a cauldron, or, or Gladiator arena. I had no escape; the only escape was through the security guard. Gregory

Like the idea of wasting others time, participant’s felt like they were a waste of space.

I had the impression from staff [in ED] that I was wasting their time and space when really, you know, they said they had genuinely sick people who should be having that bed. Fiona

Participants experienced space within the physical presence of others and reflected on their position in taking up space. The physical space of receiving care impacted the impression of safety, and bleak spaces were experienced as conveying a lack of respect for their needs.

#### Disconnected and wanting to be seen and heard (sociality)

The crisis was experienced within a wider social context, and for many participants, it was isolating, and they felt disconnected from the people around them.

I became more withdrawn and not doing all the things that I used to do. And not spending time with other people, and not talking. Andy

Other participants wanted closer contact and recognized their need for connection during crisis.

I get really sensitive in the sense that like I don’t want to be alone. And I want that physical connection. So, I’ll get really clingy. Tiffany

Shame featured in many participants’ narratives of their experience. Participants spoke of complex experiences of shame and silencing.

So then going into crisis, I definitely felt shame. Suicide already has so many layers, and then add cultural limitations around that, or cultural shame around it. It’s like the elephant in the room. Marissa

In reflection, this experience spurred a desire to be seen and heard.

I wanted to feel heard and to feel seen. Often what led me to be in crisis was that I felt invisible, or that I felt I had no purpose or that I felt misunderstood. It felt like no one, no one knows my life, no one has lived what I’ve lived through. Marissa

Crisis represented dual dimensions of feeling disconnected from others and a desire for connection. The ability to receive crisis care was dependent on relational elements, which were simultaneously fraught with feelings of shame, driving participants towards disconnection. For participants, shame is related to wanting to be seen, heard and understood.

#### Loss of meaning and purpose (project)

For many participants, crisis presented as a loss of meaning and purpose and a disconnection from what was significant in life. Many participants described the suicidality they experienced during a crisis as linked to a loss of hope and purpose. Paradoxically, many participants also shared experiences of activation as a result of crisis. Some participants described their experience as a spiritual crisis.

It feels like a spiritual crisis to me. I’m not necessarily a spiritual person, but I’ve often thought about it, like, I feel really disconnected from what’s important in living and things that bring warmth and direction. So, I mean, because I’m not a religious person, there’s not a go-to person to receive that experience and the closest thing we have to that, in secular communities, is medicalising what’s going on. Morgan

Resembling the work of Shneidman (1993), participants identified pain or wounding within their soul.

There’s a lot of pain in my soul, if that makes sense. My soul and my very being felt very much in pain. Charlie

Spirituality was related to participants’ capacity to find hope and to feel connected to something or someone that held meaning in life. Spirituality was also linked to the totality of the crisis experience, and the pain was identified within the soul. For nearly all participants, the hopelessness and embodied experience of pain felt unbearable, giving rise to feelings of suicide.

Most of the time, I was just in this extreme crisis where I was feeling like so emotionally overwhelmed. And, like, the emotional pain was just so intense. I just didn’t want to. I didn’t feel like I could keep going, like keep living with these really dark thoughts. Cathy

Crisis represented the shrinking of possibilities, where suicide was identified both as a feature and a resolution of the crisis. Suicide was described by some as a means to escape their excruciating embodied experience and felt understandable in the context of their lives. This section on spirituality shows how a phenomenological approach may help understand suicidality within the experience of crisis.

#### The experience has been transformative (project)

In the following section, we consider how a phenomenological approach also offered a counterpoint to the negative experience of crisis, represented by greater openness to renewed purpose and transformation. All participants recognized valued qualities that they attributed to crisis experiences.

I’ve learnt that my experiences were extremely meaningful, and still are when I experience crisis, and they were related to things that had happened to me in the world … You know, there’s some echoes of that stuff, probably will always bounce back from time to time. But really, my experience of healing work that continues has been quite transformative. So, I think I’m kind of impressed at how extraordinary, like, dude, you’re kind of really weird and creative. Blair

Participants recognized that the crisis was part of experiencing the fullness of life.

And I love reading Daring Greatly at the moment. I think she talks about Roosevelt in the beginning and of being in the arena. And that sort of feels like for me, you know, post-crisis. It’s like, you know. It’s worth living in the arena. And in order to live, you’ve got to be in that arena. And you’ve got to have good and bad days and richness and the opportunities and all that comes with that. Addison

Some participants identified negative crisis care responses fuelling their desire to change the system and social attitudes to suicide and mental health crises.

That’s what I found in the beginning, I used to hide it. In the beginning, I used to pretend that it didn’t happen, and I wouldn’t talk about it. And then, one day, somewhere along the line, I thought, well, if I don’t talk about it, and I don’t acknowledge it, it’s gaining power. The more that I talk about it, the more that I acknowledge it, and the more that I use it to help other people. The more that it loses its power. Leanne

A crucial component of crisis is the articulation of the tension between existential vulnerability and renewed purpose. This paradox extends the provocative task of understanding crisis within the context of spiritual pain juxtaposed with the potential for transformation. Together, the embodied experience and “tacit understanding” of crisis comes into focus using the Lifeworld approach as a form of integration through reflectively focusing on lived experience.

## Discussion

The findings uncovered a phenomenological account of mental health crisis based on sensory experience, giving insight into the embodied nature of crisis. The body and its emotions provided deeper insights into the experience of being in crisis while being in the world. Given the complexity and diversity of participant experiences, it was difficult to coalesce common embodied experiences of crisis. Considered together, a crisis was generally experienced as a tangle of overwhelming sensory experiences that negatively impacted the sense of self and capacity to be in the world. For many participants, their crisis reached a threshold, and suicide was a way of escaping the unbearable pain. Encounters with others during crisis were fraught, yet all participants recognized an inability to cope without support from others.

Previous research has characterized the intense emotions associated with crisis as pain, misery, urgency, and defeat, often with dire fear about the future (Callahan, 1998; Hendricks & Byers, 2002; Parry, 1990). Similarly, each participant described overwhelming feelings, such as fear, despair, horror, shame, and sadness. Emotions were easily accessible, and feelings coloured other aspects of crisis, reflecting the power of mood in defining experience (Todres & Todres, 2007). Emotional experiences are characteristic of crisis associated with psychosis, which reveals psychosis as a disruptive “personal crisis” involving intense shame, anxiety, confusion, and despair (Bögle & Boden, 2019; Hutchins et al., 2016). Similarly, overwhelming emotional pain is common in understanding suicide and self-injury, with feelings of helplessness, shame, fear, and guilt (Joiner, 2005; Leenaars, 1996; Shneidman, 1993).

Physically, mental health crises have been identified with somatic symptoms (APA, 2013), and somatic manifestations of crisis are prevalent in psychodynamic writings (Gupta, 2013). Importantly, physical dimensions are sometimes overlooked in mental health care (Spurrier et al., 2023), and findings support a call for a whole-person approach, beyond mental health needs alone, with consideration to the physical expression of crisis. Attention to physical needs during a crisis also opens the scope for physical comfort or other sensory modulation, which has been found to reduce distress for people during a mental health presentation in ED and decrease rates of restraint (Adams-Leask et al., 2018; Bowman & Jones, 2016).

People with mental health diagnoses often face physical health challenges that adversely affect their quality of life and risk disadvantages within healthcare systems that operate on the assumption that the mind and body are separate (Link et al., 2017). Eating disorders are a clear example of the need for integrated physical and mental health care to enrich holistic approaches (Corral‐Liria et al., 2022). The findings reassert the importance of dismantling silos in mental and physical health care to enable a more holistic approach and find ways of moving beyond the Cartesian dichotomy (Kaushik et al., 2024).

During a crisis, the sensation of time resulted in a sense of urgency that seemed to signal heightened tension within the current experience and a desire for the experience to pass. This sense of immediacy is reflected in existing literature on crisis (Reynolds, 2019) and is thought to relate to the flight or fight mechanism in response to extreme stress (Chu et al., 2022). Crisis states were also revealed in the subjective experience of physical space, with EDs contributing aversive elements. Relatedly, a recent study highlighted the subjective perception of a “safe space” during a mental health crisis as one that is not restrictive and allows for interaction with others (Andrew et al., 2023).

The disruptive quality of crisis provides a compelling catalyst for spiritual questioning and transformation. Heidegger provides an understanding of human existence that cannot be separated from a broader spiritual context (Heidegger, 1998). He wrote that there is an existential need and disruption that is created by the feeling of loss of trust and attunement to the world and others (Heidegger et al., 1962). Likewise, Todres eloquently expresses “human embodiment as the integrating ‘place’ where both human vulnerability and spiritual freedom can happen” (Todres et al., 2007, p. 124). From this orientation and in line with previous research, this study found an understanding of mental health crisis as related to spiritual struggles (Ritsher et al., 1997; Swaan, 1990). Spirituality overlaps with an existential understanding posed by van Deurzen as related to meaning and purpose (Van Deurzen, 2014). The metaphor of “soul pain” in the findings ascribes the human experience of crisis with an existential or spiritual “wound.” Research has also pointed to increasing evidence of spiritual crises involving altered states and intense emotional and psychosomatic manifestations (Grof & Grof, 2017).

Findings uncover a paradox of vulnerability and growth, indicating a non-deterministic dimension to our understanding of crisis and creating tension for crisis care to enable and not stifle growth. Therefore, crisis considered as an existential challenge allows suffering to be conceived as undesirable and difficult but as an intrinsic and essential part of human life and even potentially life-enhancing. Findings indicate the humanizing potential in understanding the purpose of crisis as both disruptive and transformational and as foundational for greater empathy and hope. The transformative potential is also noted in broader mental health literature, notably in relation to psychosis (Attard et al., 2017).

The view that crisis is transformational is in opposition to views that crisis is an intrinsically harmful, aetiological factor in creating mental illness (Eastham et al., 1970). Instead, findings reveal that the experience of a mental health crisis was a complex sensory experience enmeshed in people’s sense of self, identity, and social context. The complexity of the embodied experience evident in findings is in line with previous research showing that crisis is multifaceted and subjective (Borg et al., 2011; Gullslett et al., 2014, 2016; Hansson, 2012; Winness et al., 2010). Further, this study contradicts an understanding of mental health crisis fundamentally as a biological event or an exacerbation of a pre-existing diagnosis (Ball et al., 2005). Reducing the experience of crisis to an expression of symptoms of mental illness was not adequate. This points to the need for crisis responses to more broadly conceptualize crisis as a multifaceted sensory experience underpinned by emotional pain stemming from life challenges (Gullslett et al., 2014). This approach is supported by literature that clearly points to the need for more human dimensions to mental health care, incorporating approaches such as Open Dialogue, peer support, and psychotherapy (Stupak & Dobroczyński, 2021).

We make no claim that this understanding of crisis can be generalized to every circumstance. As with any qualitative study, it is for the reader to determine the resonance of the findings. A strength of this study is the foregrounding of lived experience. The phenomenological approach in this study made room for elaborate descriptions of deeply emotional experiences and metaphors that arose from the interviews contributing towards an embodied understanding of crisis. It is argued that phenomenology is underutilized in medicine and can be applied to narrow the gap between objective clinical assessments and subjective experience (Carel, 2011). According to Svanaeus, phenomenology not only describes but can provide first-person definitions of illness (Svenaeus, 2019). The findings raise criticism of a reductionist approach to understanding crises within psychiatry and show how a phenomenological approach can assist in rethinking existing crisis care practice.

## Conclusion

This study has emphasized the meaningfulness of the sensory experiences associated with a crisis. The emotional, physical, and cognitive elements were significant and in parallel to loss of self and feeling disconnected. These feelings were entwined and experienced as overwhelming, resulting in feelings of suicide. Given the centrality of overwhelming emotions to the crisis experience, crisis services that provide physical and emotional safety through therapeutic connection and somatic approaches may better support recovery. Similarly, holistic approaches that see a crisis as a meaningful experience and as something to be acknowledged and explored may also offer longer-term benefits by letting people discover meaning and opportunities to reconstruct their sense of self and place in the world.

## Supplementary Material

Biographical information for authors.docx

## Data Availability

The data supporting this study’s findings are available on request from the corresponding author. The data are not publicly available because they contain information that could compromise the privacy of research participants.
