# Supplementary material for: The anatomy of crisis
Source: Int J Qual Stud Health Well-being. 2024 Oct 17;19(1):2416580. doi: 10.1080/17482631.2024.2416580 (PMC11488168; doi:10.1080/17482631.2024.2416580)
Supplement: Biographical information for authors.docx [file ZQHW_A_2416580_SM5048.docx]

**Helena Roennfeldt (MSW)**

Helena Roennfeldt is a PhD candidate and researcher who specializes in lived experiences of distress and crisis. Her research focuses on mental health crisis experiences and formal mental health crisis responses. Helena has conducted extensive research on the development of the Lived Experience workforce in Australia. She holds a master’s degree in social work, Forensic Mental Health, Suicidology, and Mental Health Practice. With over 20 years of experience in the mental health sector, her academic, practical, and lived experience has provided her with a strong knowledge and skills base in qualitative research.

**Associate Professor Bridget Hamilton (PhD)**

Associate Professor Bridget Hamilton serves as the Director of the Centre for Mental Health Nursing. Her role involves overseeing a team of clinical nurse academics and consumer academics. The main objective is to enhance the skills and contributions of mental health nurses in Victoria, which in turn will benefit people who receive mental health care. As a clinical academic and leader in the public mental health sector, all her research, teaching and engagement work is carried out in collaboration with consumers and carers. Her ultimate goal is to develop service models and mental health nursing practices that are aligned with the priorities of people experiencing mental health problems and crises.

**Dr Nicole Hill (PhD)**

Dr Nicole Hill has a diverse background that includes experience in clinical, academic, research, and teaching. She has a rich history of working in acute and crisis mental health. Currently, she is a part of the Department of Social Work in the Melbourne School of Health Sciences at The University of Melbourne. Her role is multifaceted, involving lecturing in the Master of Social Work program and boosting the research profile of Field Education. As a member of the Department's Practice Research Program in Health and Mental Health, she is continuing her doctoral studies on suicide prevention by collaborating on various local and international research and training projects.

**Dr Louise Byrne (PhD)**

Dr. Louise Byrne is a Senior Research Fellow at RMIT University and an Assistant Professor Adjunct at Yale University. She is a Lived Experience researcher who specializes in workforce development and is recognized as a leading international expert in the field of Lived Experience-led research. Louise has received training in Intentional Peer Support and has worked in various Lived Experience/peer roles since 2004, including positions in the government and not-for-profit sectors. She has been in academic positions since 2009.
